# Neuronal MHC-I display in T-cell mediated neurodegeneration

**DOI:** 10.1186/1750-1326-8-S1-P38

**Published:** 2013-09-13

**Authors:** Carolina Cebrián, Fabio Zucca, Luigi Zecca, John Loike, David Sulzer

**Affiliations:** 1 Columbia University, New York, USA; 2 Italian National Research Council, Milano, Italy

## 

Parkinson’s disease (PD) and other disorders feature the degeneration of ventral midbrain (VM) catecholamine neurons. Recent data suggest that neuroinflammatory mechanisms contribute to a cascade of events leading to chronic neuronal degeneration.

In primary murine neuronal cultures, substantia nigra (SN) and locus coeruleus (LC) neurons are induced to express the major histocompatibility class I complex (MHC-I) by the proinflammatory cytokine, γ-interferon, L-DOPA, or conditioned medium from microglia exposed to α-synuclein or NM. SN DA neurons, moreover, process the foreign protein ovalbumin to an antigenic peptide that is presented by their MHC-I and triggers their specific destruction by CD8+ killer T-cells. In human postmortem samples, we find by immunolabel, mRNA profiling, and proteomic analysis that neuromelanin (NM)-containing catecholamine SN and LC neurons in adult human control and PD brains express MHC-I, often in proximity to CD8+ T-cells. These data reveal a novel inflammatory T-cell mediated neurodegenerative processes that could underlie neuronal death.

